# *Plant Physiology* welcomes Assistant Features Editors starting in 2025 and 2026

**DOI:** 10.1093/plphys/kiae661

**Published:** 2024-12-12

**Authors:** Yunde Zhao, Judy Brusslan, Mike Blatt, Mary Williams

**Affiliations:** Plant Physiology, American Society of Plant Biologists, USA; Section of Cell and Developmental Biology, University California San Diego, 9500 Gilman Drive, La Jolla, CA 92093-0116, USA; Plant Physiology, American Society of Plant Biologists, USA; Department of Biological Sciences, California State University Long Beach, Long Beach, CA 90840, USA; Plant Physiology, American Society of Plant Biologists, USA; Laboratory of Plant Physiology and Biophysics, University of Glasgow, Glasgow G12 8QQ, UK; Plant Physiology, American Society of Plant Biologists, USA

*Plant Physiology* is pleased to announce the new Assistant Features Editors (AFEs) who are joining the editorial board in 2025 and 2026 (see figures). *Plant Physiology* started the AFE program 7 years ago to help disseminate discoveries published in the journal and to train the next generation of editors and reviewers. Our AFEs are promising early-career scientists. They bring their passion for science to our journal, communicating to our readers each month some of the most exciting advances in research.

The AFEs have added substantially to the plant science community and to the journal. Their contributions have expanded our content through *News and Views* articles, blog posts, and related material highlighting content of special interest in *Plant Physiology*. They have grown professionally and will build on their experience with the journal. Many of our previous AFEs have moved onto independent academic positions after completing their terms at *Plant Physiology*. We hope to call the AFEs to serve as regular members of the editorial board when they have become more established in their careers.

We, too, have learned much from working with the AFEs these past 7 years. We have honed the way we support them in writing *News and Views* articles and have streamlined oversight through the journal online submission system. We have invited them to write profiles of more senior members of our editorial board, which we publish on our Plantae blog. AFEs are invited to attend the annual editorial board meetings of *Plant Physiology* where they can learn the intricacies of the editorial process and contribute to discussions of *Plant Physiology* policies and operation. And the AFEs work with a very experienced team of editors (Y.Z., J.B., M.B., and M.W.), receiving formal training and formative feedback on their writing and experiencing different editorial styles.

The program is designed to rotate off some AFEs each year and to add new members at the beginning of each year. Nineteen of our AFEs stepped down from the editorial board at the end of 2024, and we would like to take this opportunity to thank them all for their contributions. We would also like to welcome the new AFEs starting in January 2025 and will work alongside our seasoned crew (see below). Additionally, we have recruited a second cohort who will begin their term at the start of 2026.

So, welcome! We are thrilled to have both the new and returning AFEs with us. We want, also, to add our special thanks to the ASPB and the ASPB Publications Committee for supporting this initiative. With these new members, we are pleased to note that the expanded group reflects the broad distribution of research topics published in *Plant Physiology*.

NEW *PLANT PHYSIOLOGY* AFES (2025–2026)

- James Bradley (University of Toronto, Canada)
- Thomas Depaepe (Ghent University, Belgium)
- Laura Fernández de Uña (University of Vigo, Spain)
- María Flores-Tornero (ITQB NOVA, Portugal)
- Catherine Freed (University of Wisconsin-Madison, USA)
- Alice Gauthey (University of Birmingham, UK)
- Nilesh Gawande (Indian Institute of Technology Gandhinagar, India)
- Mamoona Khan (University of Bonn, Germany)
- Jitesh Kumar (University of Minnesota, USA)
- Neeta Lohani (Donald Danforth Plant Science Center, USA)
- Josephine Maidment (Plant Health Institute of Montpellier/Centre de Biologie Structurale, France)
- Jazmin Reyes-Hernandez (University of Copenhagen, Denmark)
- Sara Shakir (INRAE Bordeaux, France)
- Gunjan Sharma (University of Birmingham, UK)
- Avilash Singh Yadav (Cornell University, USA)
- Marcella Teixeira (Washington State University, USA)
- Guannan Wang (Stanford University, USA)
- Bo Xu (University of Adelaide, Australia)
- Ning Zhang (Shandong Agricultural University, China)

The new AFEs are joining those who started in January of 2024, listed below, who will stay on for one more year to continue writing news and views, help with the transition, and mentor the new AFEs (see https://doi.org/10.1093/plphys/kiad631).

- Burcu Alptekin (University of Wisconsin, USA)
- Kumari Billakurthi (University of Cambridge, UK)
- Pablo I. Calzadilla (Institute for Integrative Biology of the Cell [I2BC], Université Paris-Saclay, CEA, France)
- Erin Cullen (Max Planck Institute for Plant Breeding Research, Cologne, Germany)
- Prateek Jain (Elysia Bio, USA)
- Alicja Kunkowska (Sant'Anna School of Advanced Studies, Pisa, Italy)
- Maneesh Lingwan (Donald Danforth Plant Science Center, USA)
- Anna Moseler (University of Bonn, Germany)
- Sara Selma Garcia (Center for Plant Systems Biology of VIB Ghent, Belgium)
- Ritu Singh (University of California, Davis, USA)
- Chong Teng (University of California, Davis, USA)
- Héctor H. Torres-Martínez (Stanford University, USA)
- Thu Tran (Cold Spring Harbor Laboratory, USA)
- Nicola Trozzi (John Innes Centre, UK)
- Munkhtsetseg Tsednee (Agricultural Biotechnology Research Center, Academia Sinica, Taiwan)

We thank those AFEs whose term started in 2023 and who are now rotating off the program. It has been a pleasure working with you these past 2 years! See https://plantae.org/2023-plant-physiology-assistant-features-editors/.

- Dechang Cao (Institute of Botany, Chinese Academy of Sciences, Kunming, China)
- Jiawen Chen (KU Leuven, Belgium)
- Joke De Jaeger-Braet (University of Hamburg, Germany)
- Manuel Gonzalez-Fuente (Ruhr-University of Bochum, Germany)
- Henning Kirst (University of Cantabria, Spain)
- Yee-Shan Ku (Chinese University of Hong Kong, China)
- Aida Maric (University of Freiburg, Germany)
- Hannah McMillan (Duke University, USA)
- Sebastian Moreno (Sainsbury Lab Cambridge University, UK)
- Dyoni Oliveira (Center for Plant Systems Biology of VIB Ghent, Belgium)
- Lara Pereira (University of Sheffield, UK)
- Moona Rahikainen (University of Helsinki, Finland)
- Janlo Robil (University of Alberta, Canada)
- Alaeddine Safi (Center for Plant Systems Biology of VIB Ghent, Belgium)
- Maria-Angelica Sanclemente (University of Florida, USA)
- Henryk Straube (University of Copenhagen, Denmark)
- Jiaqi Sun (Shandong University, China)
- Kyle Swentowsky (Cold Spring Harbor Lab, USA)
- Ryo Yokoyama (Max Planck Institute of Molecular Plant Physiology, Germany)

Finally, we have offered positions to start in January 2026 to the people listed below.

- Matheus Bianconi (University of Toulouse, France)
- Yuzhen Fan (Australian National University, Australia)
- Alison Gill (University of Adelaide, Australia)
- Eva María Gómez Álvarez (Scuola Superiore Sant'Anna, Italy)
- Alyssa Kearly (Boyce Thompson Institute, USA)
- Praveen Khatri (Western University, Canada)
- Rose McNelly (John Innes Centre, UK)
- Arijit Mukherjee (University of North Carolina at Chapel Hill, USA)
- Lisa Oskam (Wageningen University, Netherlands)
- Jessy Silva (University of Porto, Portugal)
- Deeksha Singh (University of California Davis, USA)
- Hannah Rae Thomas (Zhejiang University, China)
- William Thomas (University of Western Australia, Australia)
- Benjamin Tremblay (The Sainsbury Lab, UK)
- Mireia Uranga (Centre for Research in Agricultural Genomics [CRAG], Spain)

**Figure:**
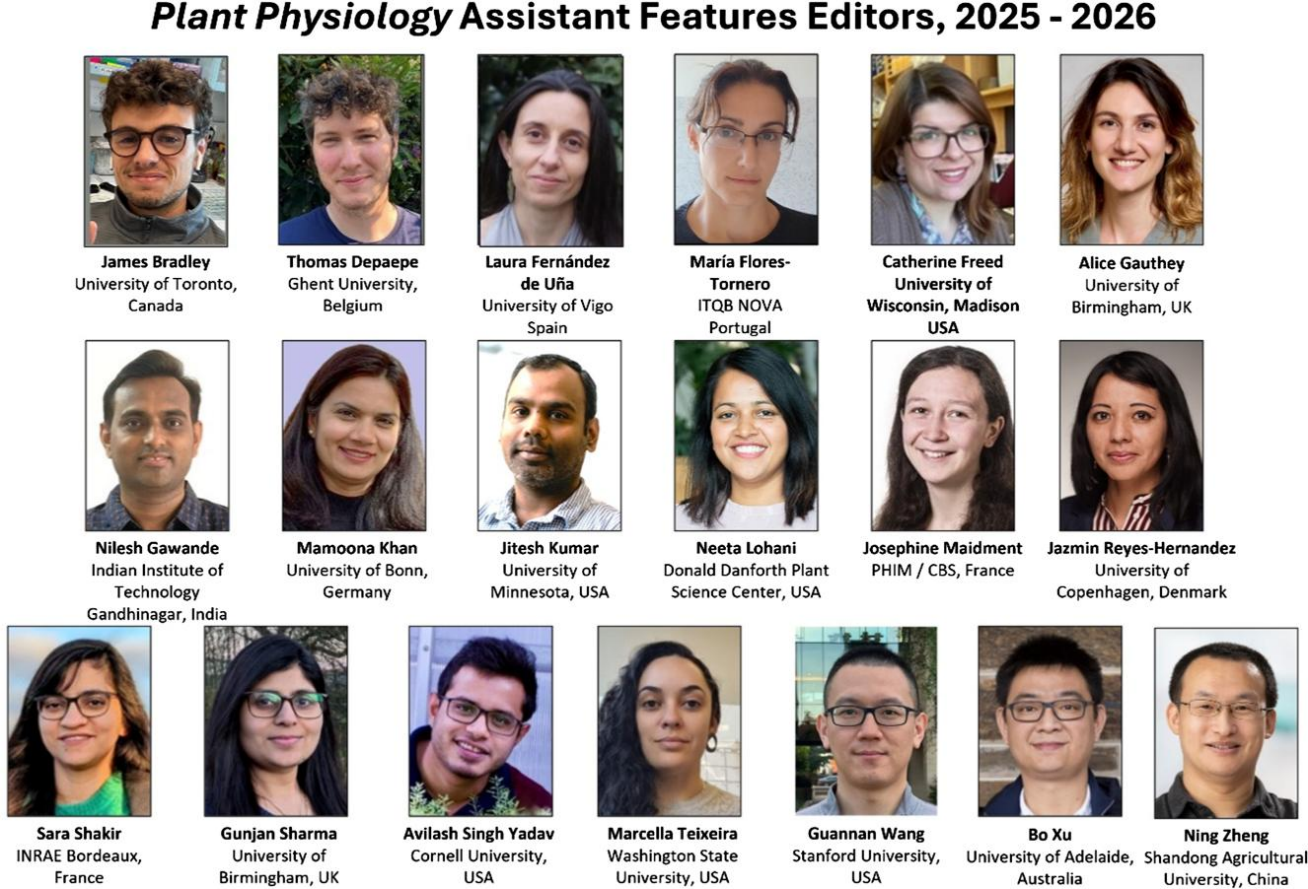


**Figure:**
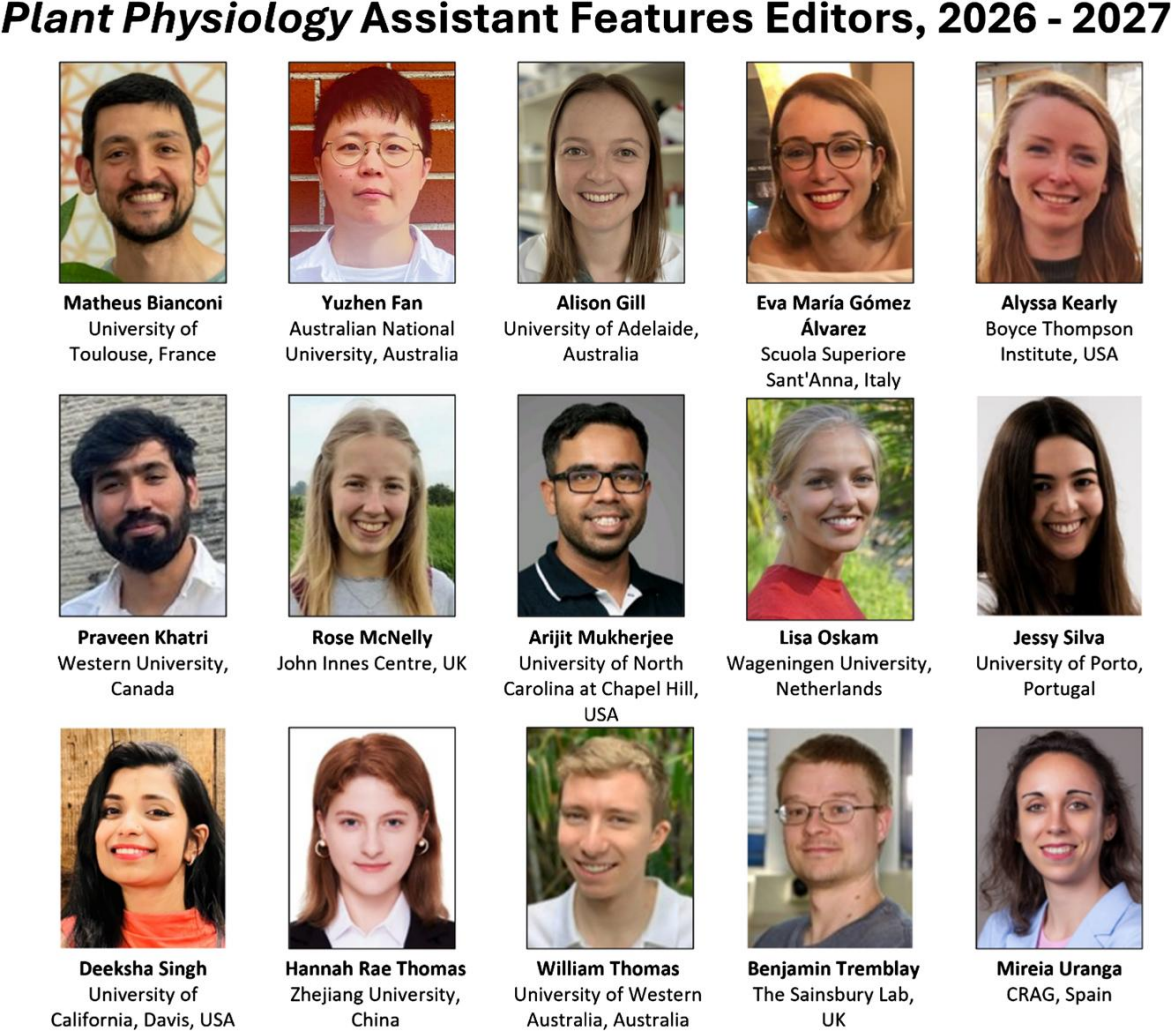


## Data Availability

No new data included in this article.

